# The role of *Pantoea stewartii* subsp. *stewartii* leucine-responsive regulatory protein (Lrp) during maize xylem growth

**DOI:** 10.1128/aem.00853-25

**Published:** 2025-06-05

**Authors:** Wilson M. Farthing, Abigail M. Heimbach, Ann M. Stevens

**Affiliations:** 1 Department of Biological Sciences, Virginia Tech 1757, Blacksburg, Virginia, USA; 2 Center for Emerging Zoonotic and Arthropod-borne Pathogens, Virginia Tech 1757, Blacksburg, Virginia, USA; Michigan State University, East Lansing, Michigan, USA

**Keywords:** corn, phytopathogen, RNA-Seq, Stewart’s wilt, transcriptome

## Abstract

The bacterium *Pantoea stewartii* subsp. *stewartii* (*Pss*) causes Stewart’s wilt disease in maize. *Pss* is introduced into maize via the corn flea beetle vector, *Chaetocnema pulicaria*, when beetle feces enter wounds created during feeding. The infection begins in the apoplast of the leaf where *Pss* causes leaf blight. Subsequently, the bacteria move to the xylem and form a biofilm, preventing water transport. This causes wilting and leads to necrosis, consequently affecting both crop yield and survival. A previous Tn-Seq experiment identified genes essential for *Pss in planta* survival. One essential gene, *lrp*, encodes the global transcription factor leucine-responsive regulatory protein (Lrp). The Lrp protein family is found across many bacterial and archaeal species where it regulates multiple critical physiological functions. In *Pss*, Lrp is known to positively control motility and capsule production, which are important for the *in planta* lifestyle and virulence of *Pss*. In this study, the genes within the *Pss* Lrp regulon were defined through bioinformatic analyses of RNA-Seq data that measured differential gene expression between wild-type *Pss* and a *∆lrp* strain grown *in planta*. Lrp was found to regulate genes involved in capsule biosynthesis and nitrogen-associated assimilation and metabolism. Biolog plates were subsequently used to link the regulatory role of Lrp with regard to *Pss* metabolism by examining the capacity of *Pss* to grow using sole carbon or nitrogen sources *in vitro*. Collectively, this work has provided insights into how *Pss* recognizes and exploits the maize xylem environment.

**IMPORTANCE:**

The bacterium *Pantoea stewartii* subsp. *stewartii* (*Pss*) causes Stewart’s wilt disease in maize when it forms a biofilm in the xylem that prevents water flow. Little is known about how *Pss* is able to colonize and grow within the maize xylem. Previous work identified the Lrp regulatory protein as being important for the survival of the bacterium inside maize. This study determined the genes whose transcription is under Lrp control and predicted the physiological functions associated with those genes to learn more about the bacterial growth inside the plant. The ability to transport and metabolize organic compounds containing nitrogen and the ability to produce capsule were found to be regulated by Lrp. Additional laboratory experiments demonstrated that Lrp also controls the metabolism of certain sole carbon and nitrogen sources. Together, these findings provide new insights into how Lrp enables *Pss* to respond to nutrient availability in the maize xylem environment.

## INTRODUCTION

*Pantoea stewartii* subspecies *stewartii* (*Pss*) is a gram-negative, yellow-pigmented proteobacterium nested within the family *Enterobacteriaceae* with other bacterial phytopathogens. *Pss* is the known causative agent of Stewart’s wilt disease in maize plants, a disease endemic to the Mid-Atlantic and Midwestern “corn belt” regions in the United States (1). Stewart’s wilt disease has minimal impact on resistant field maize cultivars, yet it continues to significantly reduce yields in susceptible field maize and more prevalently sweet and popcorn varieties (2–4). Concern about *Pss* transmission internationally via seeds has mandated a screening procedure to ensure exported seeds are not infected with *Pss* (4, 5). There is a *Pss* risk assessment regarding the entry of *Pss* in the European Union (EU) for United States (US) maize export, and more than 60 countries have quarantine restrictions on US maize exports to prevent the introduction of *Pss* overseas (6–8).

*Pss* relies on the corn flea beetle, *Chaetocnema pulicaria*, as a vector for the initial plant infection. *Pss* is inoculated into the leaf apoplast space through feeding lesions that become contaminated with beetle feces (4, 9, 10). Delivery of the *Pss* effector protein WtsE/AvrE into the plant cells by the hypersensitivity reaction/response and pathogenicity (Hrp) type III secretion system (T3SS) is essential in the early stages of maize leaf infection and for progressive colonization of the plant (10, 11). *Pss* virulence is also mediated by the production of an exopolysaccharide (EPS), called Stewartan, that facilitates spreading through the xylem, where a *Pss* biofilm forms. *Pss* gene clusters associated with EPS production are highly regulated through a quorum-sensing (QS) system that is responsive to cell density (12). At high cell density, the *Pss* QS system derepresses the expression of the *wce* (also known as *cps*) gene clusters, which are responsible for EPS production (13). In addition to EPS production, other gene clusters responsible for adhesion and motility are also under QS control (14). *Pss* biofilm formation results in the blockage of the xylem and the development of wilt symptoms. Thus, the T3SS effector (WtsE/AvrE) and EPS virulence factors act in a coordinated manner to facilitate bacterial colonization of the plant apoplast and xylem (11).

*Pss* shifts between multiple environments over its lifetime (i.e., soil environment, insect vector hindgut, maize apoplast, and maize xylem). Therefore, the bacterium must be able to sense and adapt its lifestyle to each of these differing environments for survival. A Tn-Seq analysis was performed to determine the genes that are essential for *Pss in planta* colonization and survival in the xylem (15). Analysis of the Tn-Seq data identified 486 essential genes, with 27 of these genes being transcription factors *in planta*. A subsequent reverse genetic analysis using deletion mutants to ascertain the role of several of these transcription factors led to the discovery that the transcription factor, leucine-responsive regulatory protein (Lrp), plays an important role during xylem colonization (16).

Across the *Bacteria* and *Archaea*, Lrp regulates multiple essential processes, including amino acid metabolism, pili synthesis, and additional cellular functions (17). More specifically, Lrp regulates the expression of virulence factors in other related plant pathogens, such as *Erwinia* and *Xanthomonas* (18, 19), with the absence of Lrp leading to a decrease in virulence. In *Pss*, Lrp is known to positively control motility and capsule production that are important for the *in planta* lifestyle and virulence of *Pss* (16). The predominant focus of Lrp research has been on *in vitro* experiments (20), while *in vivo* function remains a poorly understood subject.

In this study, RNA-Seq was used to determine significant, differential gene expression between the wild-type *Pss* and *∆lrp* mutant strains when grown *in planta* within the xylem compartment to better understand the role of Lrp during *Pss* growth in the xylem. It was hypothesized that a determination of the Lrp regulon would reveal additional downstream genes important for *Pss* survival in the xylem. A complementary analysis of *Pss* growth *in vitro* using Biolog plates has linked Lrp with the metabolism of sole carbon or nitrogen sources. These studies have provided insights into the growth requirements of *Pss* in maize and may have implications for other xylem-dwelling bacterial phytopathogens.

## RESULTS

### Differentially expressed Lrp regulon genes discovered through RNA-Seq

**Fig 1:**
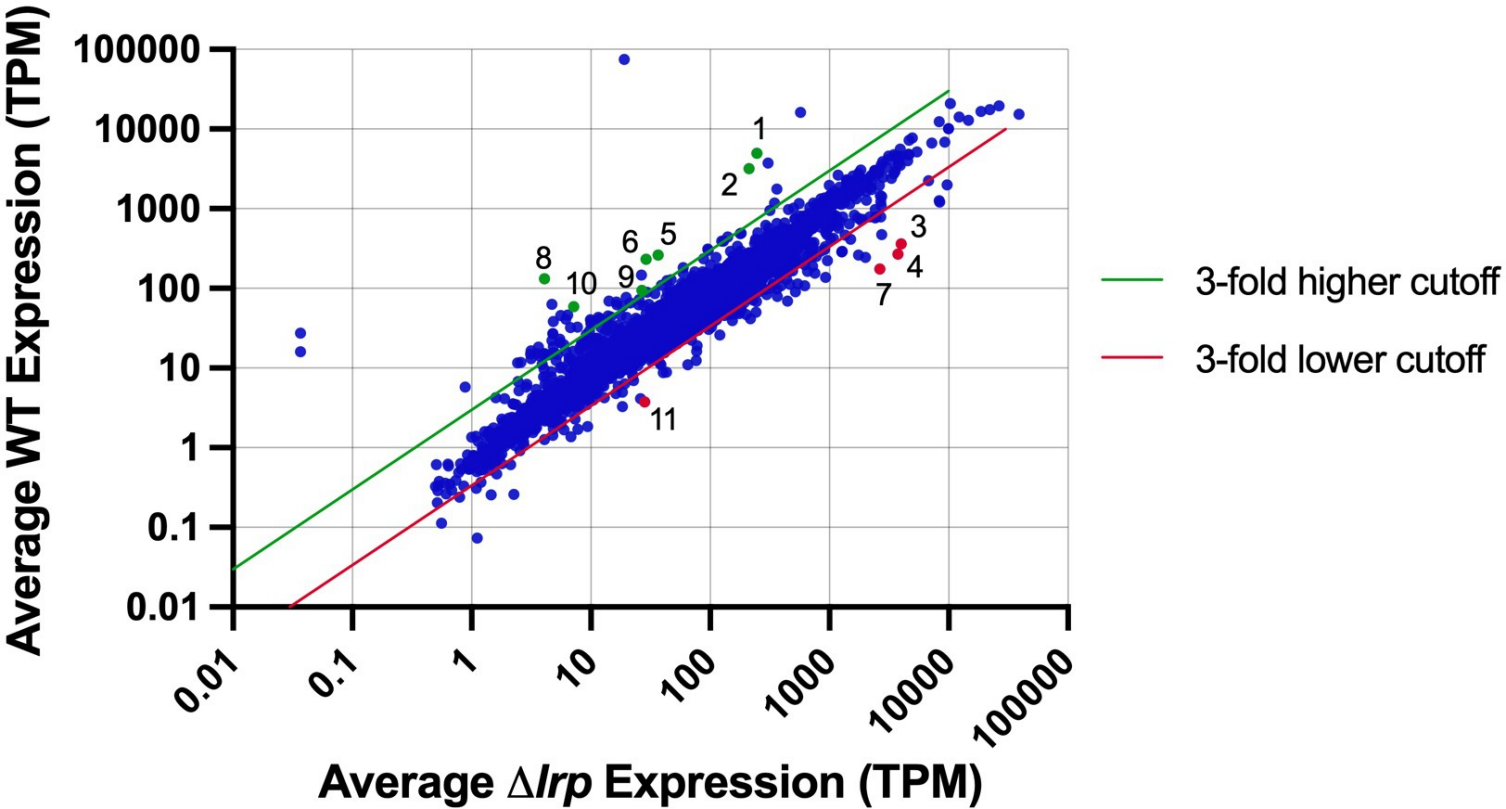
Differential mRNA expression *in planta*. Whole transcriptome data (average, normalized TPM, and bulk alignment) of *Pss* wild-type DC283 and *∆lrp* strains grown in triplicate *in planta*. Blue filled circles represent each gene. Green and red lines represent threefold higher or lower expression ratio cutoff, respectively. Any circle that falls outside of the cutoff lines is considered upregulated (green) or downregulated (red) *in planta*. The 11 genes initially selected for validation through qRT-PCR are represented as numbered green-filled (upregulated) or red-filled (downregulated) circles associated with the following encoded gene function: 1, lysozyme; 2, putative holin; 3, glycoside hydrolase family 10 protein; 4, N-acetylmuramoyl-L-alanine amidase; 5, ATP-binding cassette domain-containing protein; 6, Nac; 7, hypothetical protein; 8, YjbF family lipoprotein; 9, AceB; 10, RutA; and 11, CheR.

RNA-Seq was performed on *Pss* DC283 wild-type and *∆lrp* strain RNA extracted from *in planta*-grown cells, in experimental triplicate, to determine differential gene expression during growth in the maize xylem. RNA-Seq yielded between 50 and 76 million raw 100 bp length reads per sample that were aligned to the DC283 reference genome ([21]; Fig. 1). The normalized transcripts per million (TPM) counts for each experimental sample were calculated and averaged between replicates. Then, the averaged, normalized TPM counts were used to determine differential expression between the DC283 wild-type and *∆lrp* strains. In this manner, the genes most differentially expressed between the *Pss* DC283 wild-type and *∆lrp* strains grown *in planta* were identified. Using the criteria of a minimum of threefold differential expression and a TPM 100 count cutoff, 77 differentially expressed genes were identified from the bulk alignment. If the selection criteria were less stringent and only threefold or more differential expression was required with no TPM cutoff parameter, then overall 154 total differentially expressed genes were identified (Table S1). Eleven genes were selected for additional analysis that met the inclusion criteria of threefold or greater difference in the expression between the wild-type and the *∆lrp* mutant strains and a TPM count of 100 or greater, with the exception of the *cheR* and *rutA* coding sequences (Fig. 1 and Table 1). These two genes were included due to their greater than threefold differential expression and their location adjacent to multiple other coding sequences with significant differential expression, potentially signifying a gene cluster/operon.

**TABLE 1:** Bulk and individual alignment differential gene expression from RNA-Seq data for the *Pss* DC283 versus *∆lrp* reads of select genes

Locus tag	Gene	Annotation	Type of regulation^*a*^	Bulk alignment		Individual alignment	
				Expression ratio^*b*^	*P*-value	Expression ratio^*b*^	*P*-value
DSJ_RS03065	*yjbF*	YjbF family lipoprotein coding sequence (CDS)	A	32.54	0.042	31.55	0.043
DSJ_RS24935		Lysozyme CDS	A	20.05	7.47E-05	19.20	0.00015
DSJ_RS24945		Putative holin CDS	A	15.02	3.49E-05	14.40	0.00011
DSJ_RS25825	*rutA*	rutA CDS	A	8.25	0.064	14.05	0.041
DSJ_RS11700	*nac*	nac CDS	A	8.05	0.063	7.90	0.065
DSJ_RS08675		ATP-binding cassette domain-containing protein CDS	A	7.19	0.016	7.11	0.017
DSJ_RS03005	*aceB*	aceB CDS	A	3.53	0.16	3.43	0.16
DSJ_RS15630	*cheR*	cheR CDS	R	0.13 (7.69)	0.0015	0.13 (7.69)	0.0015
DSJ_RS23920		Glycoside hydrolase family 10 protein CDS	R	0.09 (11.11)	0.035	0.16 (6.25)	0.015
DSJ_RS23915		N-acetylmuramoyl-L-alanine amidase CDS	R	0.07 (14.29)	0.069	0.13 (7.69)	0.045
DSJ_RS13940		Hypothetical protein CDS	R	0.07 (14.29)	0.066	0.06 (16.67)	0.0019
DSJ_RS00025	*gyrB*	DNA gyrase subunit B	Control	0.97	0.59	0.94	0.21

^*a*^ A, activated or R, repressed genes *in planta.*

^*b*^ Expression ratio and *P*-value were calculated manually using the averaged, normalized TPM values of three DC283 *in planta* samples and three *∆lrp in planta* samples.

Gene choice was also driven by putative biological function annotations. DSJ_RS03065 (*yjbF*) is one of multiple genes found in the *yjb* operon essential for EPS production, further supporting the role of Lrp in regulating biofilm production (16, 22). The *yjb* genes have previously been shown to belong to the Rcs regulon (22). DSJ_RS11700 (*nac*) is known to be important in nitrogen assimilation control in *Escherichia coli* (23). Nac was one of the transcription factors found to be important during *in planta* growth (15, 16). DSJ_RS03005 (*aceB*) encodes malate synthase, which is an enzyme essential for the glyoxylate shunt pathway and involved in stress response (24). DSJ_RS25825 (*rutA*), along with other *rut* genes found on the plasmid represented by sequences in NZ_CP017591, plays a role in pyrimidine catabolism, serving as a nitrogen source in *E. coli* (25). DSJ_RS08675 (an ATP-binding cassette domain coding sequence [CDS]) is closely related to an ABC-type branched chain amino acid transporter in another *Pantoea* species (26). The remaining genes of interest (lysozyme CDS, putative holin CDS, *cheR,* glycoside hydrolase family 10 protein CDS, N-acetylmuramoyl-L-alanine amidase CDS, and hypothetical protein CDS) were chosen mainly due to their high magnitude of differential expression.

RNA-Seq data were aligned to the DC283 reference genome not only by the bulk alignment but also by an individual contig alignment (Table 1) technique. The bulk alignment was used to prevent overrepresentation of read matches for duplicate coding sequences on the 12 separate contigs (21). The two different alignment assemblies were compared to confirm likeness by matching the number of sequences, percent pairwise identity, and percent GC content, and they were found to be identical. After the calculation of differential expression for both the individual and bulk alignment assemblies, all 11 initially selected coding sequences of interest continued to meet the initial criteria of threefold or greater differential expression (Table 1). Furthermore, expression ratios of individual alignment assemblies and bulk alignment assemblies for the 11 genes remained similar, except for the expression ratio of *rutA* (Table 1), which had an increased expression ratio for the individual alignment (increase from 8.25 to 14). The majority of the remaining *rut* operon genes were also found to meet the inclusion criteria for individual alignment, likely due to an increase in their TPM (Table S2). Overall, the TPMs for all coding sequences were higher in the individual alignment assemblies compared to the bulk alignment. The number of coding sequences meeting the threefold differential expression plus TPM 100 count cutoff selection criteria was 69 in the individual alignment. Overall, there were 122 total genes with threefold or more differential expression without the TPM 100 count cutoff. These numbers for the individual alignments are slightly lower than those for the bulk alignment (Table S2 versus Table S1). While somewhat distinctive sets of differentially expressed genes were identified by the two approaches, the 11 genes of interest were identified using both methods (Fig. S1).

### DESeq2 analysis

DESeq2 analysis of the RNA-Seq data was also performed to provide differential expression confidence values between the wild-type and the *∆lrp* mutant strains for the 11 genes of interest (Table 2). Differential expression confidence values are another method of displaying significant differential expression by using the negative base 10 log of the adjusted *P*-values. All 11 genes have absolute confidence values greater than two (correlated with a *P*-value of 0.01). Negative confidence values signify genes upregulated in the wild type, while positive confidence values signify genes upregulated in the mutant. Overall, DESeq2 identified 340 genes with significant differential expression based on the differential expression confidence values, having a cutoff value of at least three (Table S3).

**TABLE 2:** DESeq2 analysis of differential expression confidence values for the *in planta* RNA-Seq data comparing the *Pss* DC283 and *∆lrp* reads

Locus tag	Gene	Annotation	Type of regulation^*a*^	Differential expression confidence value^*b*^	*P*-value
DSJ_RS03065	*yjbF*	YjbF family lipoprotein CDS	A	−38.47	2.31E-42
DSJ_RS24935		Lysozyme CDS	A	−143.91	8.46E-147
DSJ_RS24945		Putative holin CDS	A	−136.28	7.28E-139
DSJ_RS25825	*rutA*	rutA CDS	A	−8.95	2.63E-11
DSJ_RS11700	*nac*	nac CDS	A	−12.91	9.85E-16
DSJ_RS08675		ATP-binding cassette domain-containing protein CDS	A	−12.85	1.16E-15
DSJ_RS03005	*aceB*	aceB CDS	A	−4.22	2.28E-06
DSJ_RS15630	*cheR*	cheR CDS	R	24.42	5.16E-28
DSJ_RS23920		Glycoside hydrolase family 10 protein CDS	R	13.15	1.96E-15
DSJ_RS23915		N-acetylmuramoyl-L-alanine amidase CDS	R	10.46	1.92E-12
DSJ_RS13940		Hypothetical protein CDS	R	52.20	2.88E-56
DSJ_RS00025	*gyrB*	DNA gyrase subunit B	Control	−0.17	0.40

^*a*^ A, activated or R, repressed gene *in planta.*

^*b*^ DE-Seq2 differential expression analysis completed using the same read sets and individual read alignment technique. Negative differential expression confidence values correlate with upregulated genes, while positive values correlate with downregulated genes.

### Independent validation of RNA-Seq via qRT-PCR

**Fig 2:**
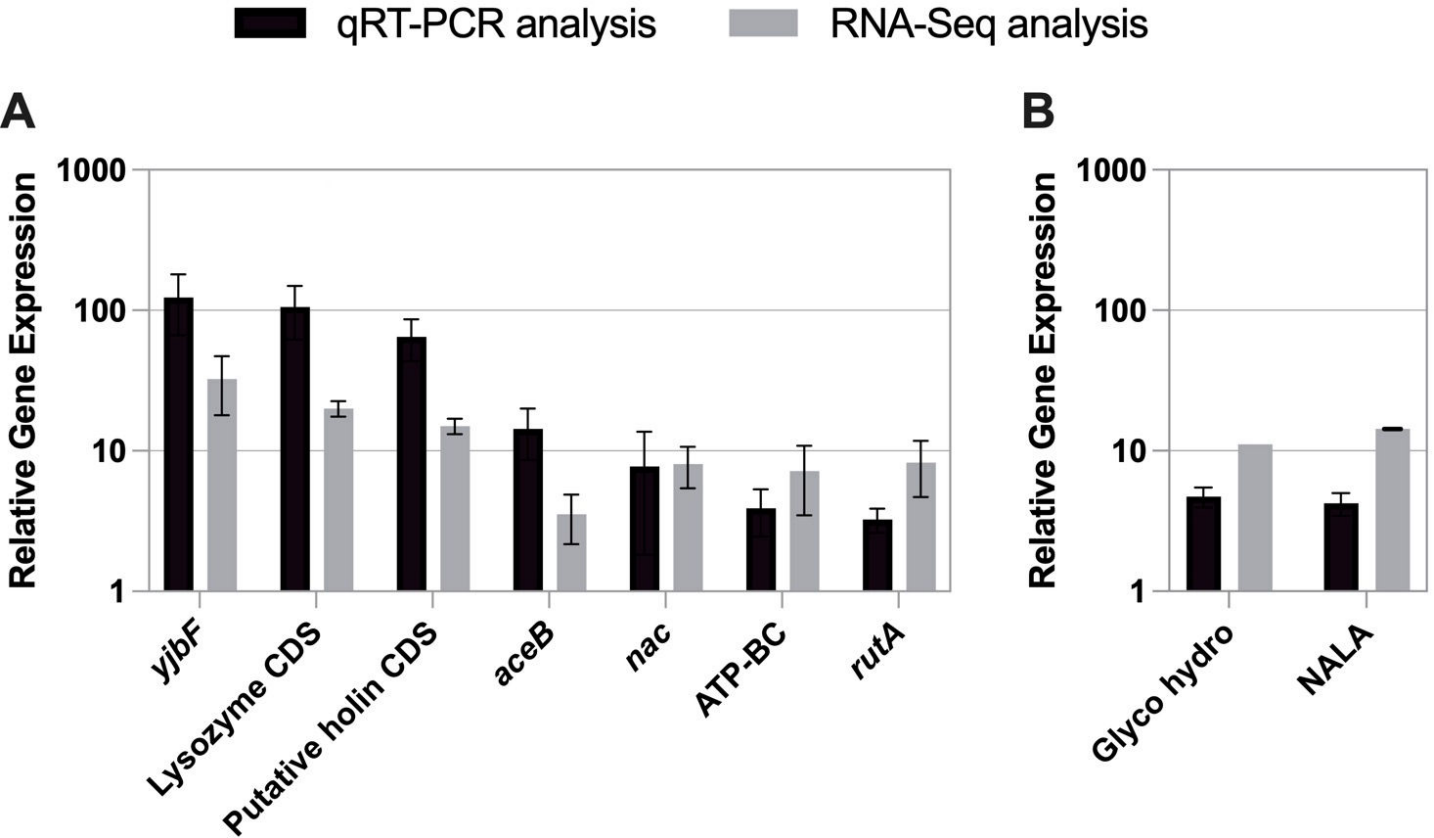
Relative gene expression from the RNA-Seq and qRT-PCR data. qRT-PCR analysis results (black) and RNA-Seq analysis results (gray) for the nine indicated genes (ATP-binding cassette domain-containing protein CDS abbreviated ATP-BC; glycoside hydrolase family 10 protein CDS abbreviated Glyco hydro; and N-acetylmuramoyl-L-alanine amidase CDS abbreviated NALA). (**A**) Fold activation or (**B**) repression is shown on a logarithmic scale. RNA-Seq and qRT-PCR results are the average of three experimental samples with the qRT-PCR analyzed in triplicate. The *gyrB* gene was used as a reference for normalization of the qRT-PCR results. Bars represent standard error (*n* = 3).

Differential expression of the 11 genes of interest (Fig. 1; Tables 1 and 2) was validated through quantitative reverse transcription PCR (qRT-PCR) using a second independent set of RNA samples. Primer sets for 9 of the 11 genes of interest were successfully optimized, and the qRT-PCR results were found to match the trends of the RNA-Seq data analysis (Fig. 2). The housekeeping control gene *gyrB* was chosen for normalization based on previous work (27), and stable expression in both the DC283 and ∆*lrp* strains was confirmed in this study. The absolute expression values for the RNA-Seq analysis and qRT-PCR were not identical; however, the trends strongly support the validity of the RNA-Seq data set.

### Protein comparisons

The amino acid sequences of the polypeptides encoded by the 11 *Pss* genes of interest were first analyzed through NCBI BLASTp alignments (Table S4). The YjbF, RutA, Nac, ABC-binding cassette domain containing protein, AceB, CheR, and N-acetylmuramoyl-L-alanine amidase CDSs all had high percent identity (100%–88%) and query coverage (100%–97%) with their respective top 10 NCBI BLASTp alignments. The glycoside hydrolase CDS had a slightly lower percent identity (93%–80%) and query coverage (100%–96%). Lysozyme, putative holin, and hypothetical protein all had a lower percent identity (65%–39%) and query coverage (99%–80%). Nevertheless, the protein comparisons for each of the select genes provided initial support for potential function.

Most of the selected coding sequences shared close similarity to proteins associated with bacteria in the *Enterobacteriaceae* family, predominantly other *Pantoea* spp. This supports the idea that these genes are highly conserved across *Pantoea* spp. Although the lysozyme and putative holin-coding sequences on the NZ_CP017591 plasmid and the hypothetical protein on the main chromosome had low identity with other proteins, the best matches were still associated with the bacteria in the *Enterobacteriaceae* family, such as *Erwinia* and *Serratia*. These results suggest that the lysozyme and holin genes may be specific to our *Pantoea* DC283 strain or that they are potentially associated with a remnant prophage.

A second bioinformatics approach to ascertain protein function was completed by performing an AlphaFold analysis (28, 29) using the amino acid sequence encoded by the 11 genes of interest to obtain the predicted protein structures. The predicted structures were then analyzed through the Dali protein server (30) to identify the top 10 matches (Table S5). Overall, the Dali protein matches supported the BLASTp analysis with regard to the predicted protein functions. In addition, this second approach helped to refine the proposed function of the hypothetical protein to that of a MARR family transcription factor. Furthermore, the structure-based analysis revealed similarities to proteins originating from a more diverse group of organisms than the initial BLASTp alignment.

### Gene Ontology analysis

**Fig 3:**
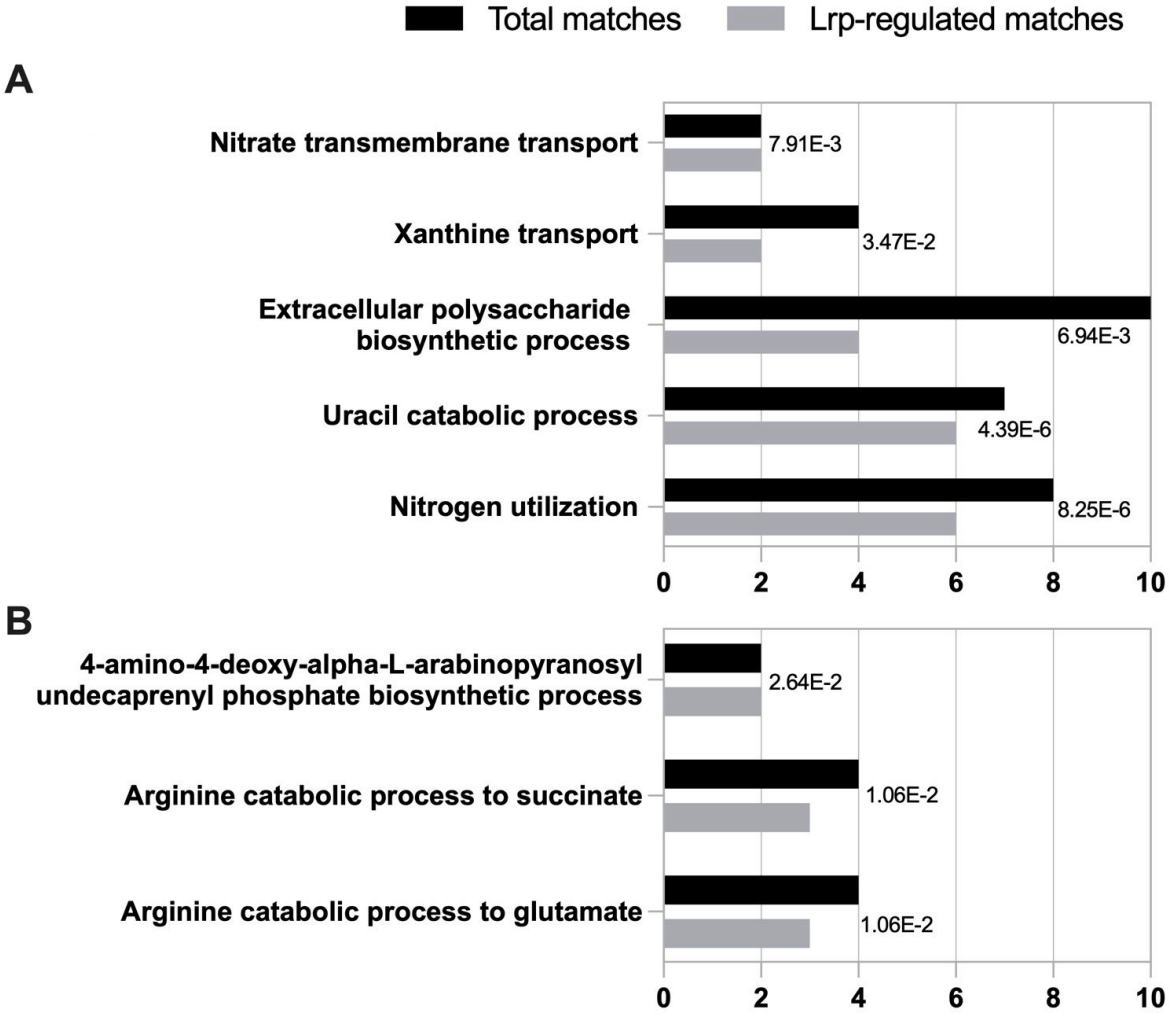
Gene Ontology analysis groups of biological process significantly (**A**) activated or (**B**) repressed by Lrp. Total number of matches in the *Pss* genome (black) compared to Lrp-regulated gene matches (gray) with associated biological process. Groups shown had at least two or more genes assigned to the same biological process group and a maximum *P*-value of 0.05 after false discovery rate adjustment, indicated to the right of each process.

Gene Ontology (GO) analysis was used to identify the classes of genes associated with bulk alignment differential gene expressions (Table S1). There were 59 annotated genes for both the Lrp-related activated and repressed genes (118 total) that were able to be given GO designations (Table S6). The most significantly activated and repressed biological processes (GO biological processes with two or more associated genes and an FDR adjusted *P*-value of 0.05 or less) were identified (Fig. 3A and B, respectively). The GO analysis identified five different groups of genes (nitrogen utilization, uracil catabolic process, EPS biosynthesis process, xanthine transport, and nitrate transmembrane transport) under the biological process hierarchy associated with Lrp-related activation and three different groups of genes (arginine catabolic process to glutamate, arginine catabolic process to succinate, and 4-amino-4-deoxy-alpha-L-arabinopyranosyl undecaprenyl phosphate biosynthetic process) associated with Lrp-related repression (Fig. 3).

### Ability of *Pss* to metabolize sole carbon and nitrogen sources *in vitro*

**Fig 4:**
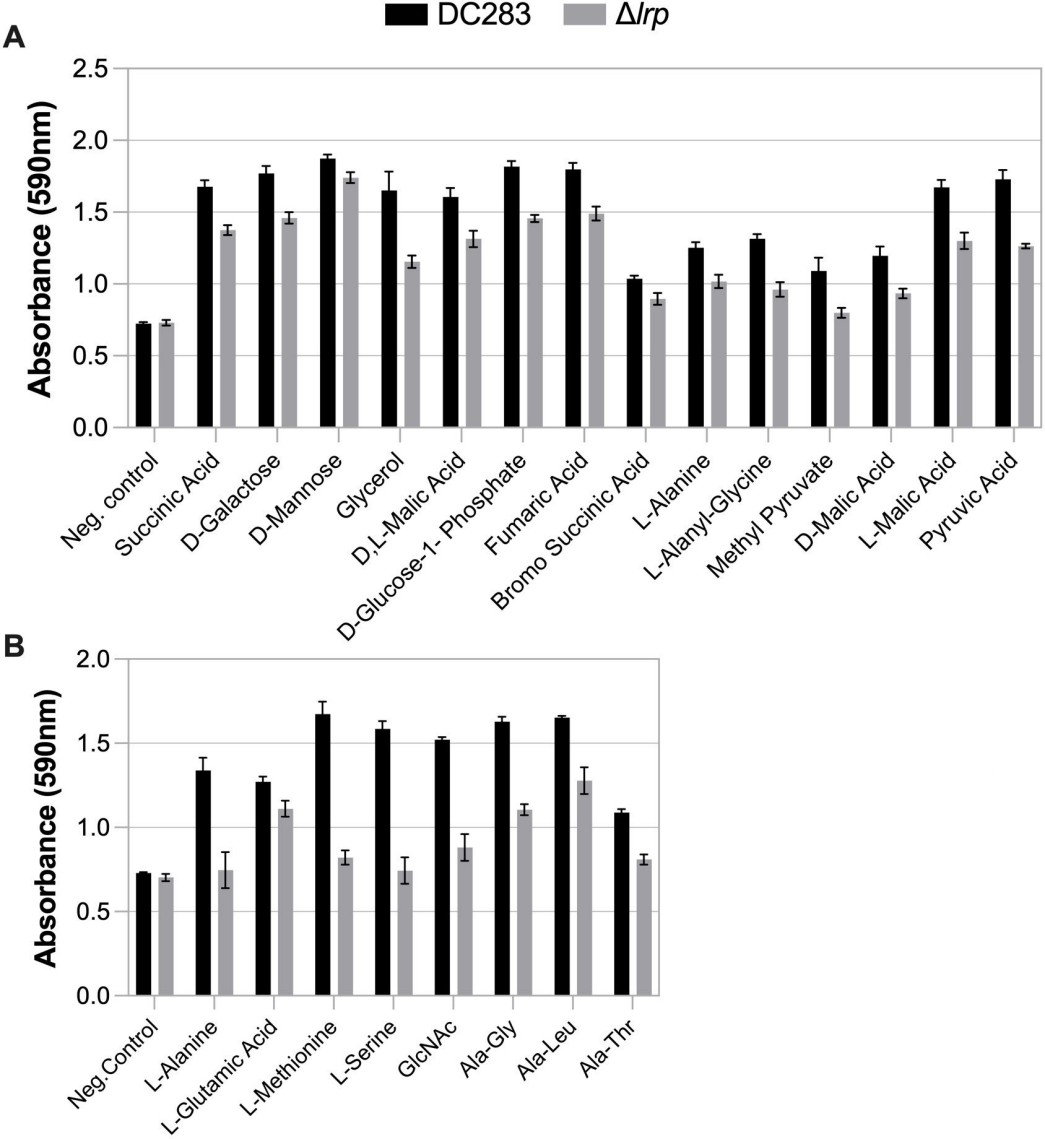
Growth of *Pss* wild-type DC283 and a *∆lrp* strain on sole sources of (**A**) carbon or (**B**) nitrogen as measured on Biolog plates. Wild-type *Pss* data are shown in black, and *∆lrp* data are shown in gray. Only those compounds that permit both significantly more growth of wild-type *Pss* versus the negative control (*P* < 0.05) and significantly less growth of the *lrp* mutant versus the wild type (*P* < 0.05) are presented. Bars represent standard error (*n* = 3).

Biolog plates were used to measure and compare *Pss* DC283 and the Δ*lrp* strain growth after 24 h on sole carbon (PM1 microplate) and nitrogen (PM3 microplate) sources, as indicated by the relative absorbance (590 nm) of tetrazolium dye. First, a *t*-test was used to determine where wild-type DC283 growth for a given substrate was significantly higher compared to the negative control. Then, only for those substrates that the wild type was capable of metabolizing, a second *t*-test was performed to compare the growth of DC283 to the Δ*lrp* strain to identify significantly reduced growth rates in the deletion strain (Fig. 4).

Out of the 95 carbon sources on the PM1 microplate, there were 41 that DC283 metabolized significantly better than the negative control (Table S7), and of these, 14 were metabolized by Δ*lrp* significantly less than the wild type (Fig. 4A). Thus, 14 carbon sources appear to involve Lrp regulation of metabolism: succinic acid, D-galactose, D-mannose, glycerol, D, L-malic acid, D-glucose-1-phosphate, fumaric acid, bromo succinic acid, L-alanine, L-alanyl-glycine, methyl pyruvate, D-malic acid, L-malic acid, and pyruvic acid (Fig. 4A). Some of these findings confirmed previous work using a defined medium to establish the metabolic capabilities of the bacterium. Malate, succinate, glycerol, fumarate, and pyruvate were sole carbon sources highly abundant in the maize apoplast, and *Pss* grew efficiently on them *in vitro* (10). L-malic acid, fumaric acid, succinic acid, and pyruvic acid are important compounds in the tricarboxylic acid cycle. D-galactose, D-mannose, glycerol, and D-glucose-1-phosphate are other carbohydrates that could be readily metabolized by *Pss* when Lrp is active. L-alanine and L-alanyl-glycine are associated with amino acid metabolic pathways and are important sources for both carbon and nitrogen.

Out of the 95 nitrogen sources on the PM3 microplates, there were 35 that DC283 metabolized significantly better than the negative control (Table S7), and eight of those were used significantly less in the Δ*lrp* strain than the wild type (Fig. 4B). Eight nitrogen sources appear to involve Lrp regulation of metabolism: L-alanine, L-glutamic acid, L-methionine, L-serine, N-acetyl-D-glucosamine, Ala-Gly, Ala-Leu, and Ala-Thr (Fig. 4B). Glutamate, alanine, and serine were abundant nitrogen sources in the apoplast that *Pss* efficiently utilized as sole nitrogen sources (10). Thus, specific organic acids, sugars, and amino acids can be used as nutrient sources by *Pss* DC283 *in vitro* (Fig. 4). By comparing Δ*lrp* carbon and nitrogen utilization patterns to DC283, possible Lrp-dependent metabolic processes were identified.

## DISCUSSION

Phytopathogens that colonize the vascular systems of plants tend to be distinguished by those inhabiting either the phloem or the xylem. Some xylem-dwelling phytopathogens, like *Pss*, first enter the plant in a continuous extracellular space known as the mesophyll apoplast, including the space between the plant cell wall and plasma membrane. These pathogens eventually enter and subsequently colonize the xylem through a poorly defined mechanism. Fluctuating xylem fluid flow, plant defenses, and scarce nutrients are thought to make the plant xylem a relatively inhospitable environment for bacteria (31).

Previously, transcriptome data of *Pss* grown *in planta* versus *in vitro* in a Luria-Bertani (LB) culture identified genes significantly upregulated during *in planta* growth (27). The largest groups of upregulated genes encoded nutrient transporters for amino acids, sugars, and other compounds. Genes encoding transcriptional regulators associated with these transporters, including *nac* involved in nitrogen assimilation control, were also upregulated (27). Other upregulated genes encoded functions associated with oxidation and reduction and included some related to oxidative stress (e.g., *cytD* encoding cytochrome oxidase) and fatty acid metabolism (e.g., *aceB* encoding malate synthase). A Tn-Seq study subsequently discovered multiple genes essential for *Pss in planta* growth and survival (15), including the global transcription factor, Lrp. Lrp was found to regulate motility and capsule production in *Pss*, factors critical for in *planta* virulence (16).

In this study, RNA-Seq was used to analyze the transcriptome of *Pss* wild type and an isogenic ∆*lrp* mutant strain during monoculture *in planta* growth to identify the Lrp regulon. Comparison of the RNA-Seq data sets using both bulk and individual alignment strategies allowed for the determination of differential gene expression (threefold or greater) between the two strains. DESeq2 was also used to calculate confidence values to further validate differential gene expression by a different approach. qRT-PCR analysis of nine select genes was performed as an independent method to validate the RNA-Seq data using a second set of RNA samples. Finally, GO analysis highlighted several biological processes as being key physiological outputs putatively under Lrp control that are necessary for *in planta* colonization and survival in the xylem.

The xylem is a difficult environment for phytopathogens to colonize. Vascular pathogens able to penetrate the xylem barriers must overcome a constantly fluctuating flow of xylem fluid. When bacteria are sensed by the host, plant physical barriers are reinforced and toxic defense compounds are secreted into the nutrient-poor xylem fluid (31). There are a limited number of vascular bacterial pathogens, but interestingly, most of these pathogens have evolved to inhabit and thrive in the xylem (9, 31, 32). Previous research shows that *Pss* is able to influence the host metabolism and extract available nutrients during mesophyll apoplast infection through its type III secretion system and the WtsE effector (10, 33). It remains unknown if *Pss* also disrupts the host plant metabolism during xylem infection, although WtsE does play an undefined role in the xylem (34). In some studies, xylem fluid has been found to contain low amounts of carbohydrates, with the predominant source of potential nutrients thought to be nitrogen-containing compounds, like amino acids (35–38).

Fifty-nine Lrp-controlled genes with threefold or greater activation (Table S6) were grouped through GO analysis based on their predicted functions. Five GO biological processes were found to be significantly activated by Lrp: nitrogen utilization, uracil catabolic process, extracellular polysaccharide biosynthetic process, xanthine transport, and nitrate transmembrane transport. Of these, the biological processes with the greatest number of Lrp-activated annotated genes were those involved in nitrogen utilization and uracil catabolic processes. All six genes encode proteins in the Rut pathway, which processes pyrimidines (uracil, thymine, and cytosine) to be utilized as a usable nitrogen source (39), were identified. The six identified genes (*rutFEDCBA*) are located in an operon on the NZ_CP017591 plasmid with *rutG* as the first gene in the operon. In *E. coli,* the Rut pathway is one of three pyrimidine catabolic pathways, and it is activated by the nitrogen regulatory protein C (NtrC) (25, 39).

Another *Pss* upregulated gene involved in the utilization of nitrogen encodes the Nac (nitrogen assimilation control) transcriptional regulator. In *Klebsiella aerogenes, Klebsiella pneumoniae,* and *E. coli*; this gene was found to be activated by the nitrogen regulatory system (Ntr) under glutamine-scarce conditions (40). *E. coli* Nac regulates histidine, urea, and proline catabolic pathways, while repressing ammonia assimilation pathways and genes like glutamine synthase and glutamine dehydrogenase (23, 40). In *Pss,* Nac was identified as an essential gene through Tn-Seq, but a deletion strain exhibited no obvious phenotype (16); perhaps it is important for nutrient acquisition from amino acids present in xylem fluid.

Upregulation of the gene *aceB*, which encodes the glyoxylate cycle enzyme malate synthase, suggests that alternative carbon sources may be present in the xylem. The glyoxylate cycle is a tricarboxylic acid pathway shunt that allows growth using acetate and fatty acids as a sole carbon source and net carbon accumulation from acetyl-CoA (41). Interestingly, there is evidence that malate synthase helps improve survival during oxidative stress due to the metabolism of glyoxylate (24). Plants are known to secrete reactive oxygen species in response to bacterial infection (9, 32), and genes involved in oxidative stress response were one of the biological processes most upregulated during *Pss* grown *in planta* (27).

The next largest group of Lrp-regulated annotated genes was associated with the activation of the EPS biological process. EPS production is the major virulence factor used by *Pss* during late-stage xylem infection and the culprit for the characteristics of Stewart’s wilt disease (4, 42). EPS is an essential virulence factor that provides protection against harsh conditions and plant defense, while anchoring the bacteria to counteract the flow of xylem fluid (4, 31). The *yjbHGFE* genes were all located adjacent to each other in an operon on the *Pss* main chromosome. In *Erwinia amylovora* (a phytopathogen that causes fire blight disease), Lrp has also been described as a virulence regulator associated with the control of EPS production (19). In *Pss*, EPS production is regulated by the EsaR/EsaI quorum-sensing system and the Rcs two-component regulatory system (43). Lrp appears to also be involved in a mechanism of coordinated activation of the *yjb* operon and the essential process of EPS production during *in planta* growth. Previous research found that the *Pss ∆lrp* strain showed reduced capsule formation and motility along with lower virulence for inoculated maize plants (16).

In the *Pss* Lrp regulon, the xanthine transport biological process was associated with the activated genes for *rutG* (the first gene in the *rut* operon as described above) and a purine permease (WP_006121072.1; found on the main chromosome). The *rutG* gene likely encodes a putative pyrimidine transporter based on its function in *E. coli* (39). Interestingly, *rutG* has been grouped in *Pss* with the transport of a purine base, not a pyrimidine. Purine permeases transport xanthine and other purine-like compounds (44). Across many bacterial genera, purines are used as carbon, nitrogen, and energy sources (44–46). Interestingly, plants secrete purine-like compounds known as cytokinins in response to a wide array of biotic and abiotic factors (47, 48). Perhaps *Pss* is capable of utilizing purine-like compounds, like cytokinins, as a minor resource when growing in the xylem.

The nitrate transmembrane transport biological process was grouped with the ABC transporter ATP-binding protein (WP_006118921.1) and *ntrB*. These two genes are adjacent to each other on the main chromosome. In many gram-negative bacteria, the general nitrogen regulatory system (Ntr) controls nitrate transport and assimilation (49). The two-component system involving the sensory kinase, NtrB, and response regulator, NtrC, is responsive to ammonia and glutamine levels (49). An activated, putative nitrate regulatory protein CDS (WP_006118918.1) is found upstream and adjacent to *ntrB* on the *Pss* main chromosome and is suspected to encode NtrC. In addition, the ABC nitrate transporter ATP-binding protein (WP_006118921.1) and two annotated, activated genes, a nitrite reductase, *nirB*, and nitrate reductase, are located adjacent to and downstream of *ntrB*. Finally, a gene upregulated by Lrp encoding an ATP-binding cassette domain-containing protein (WP_033737785.1) was found to have GO processes associated with branched chain amino acid (BCAA) transport. BCAAs consist of leucine and isoleucine, important signaling molecules for Lrp (20).

The GO analysis also provided groupings for the 59 annotated genes related to threefold or greater repression by Lrp (Table S6). Three annotated genes, *astADB*, are associated with both arginine catabolic processes, converting arginine to glutamate and to succinate, and are located adjacent to each other on the *Pss* main chromosome. In *K. aerogenes* and *E. coli*, the arginine succinyl-transferase pathway (encoded by the *astCADBE* operon) converts arginine and aspartate to glutamate and succinate in nitrogen-limited conditions (50). Interestingly, there is a repressed aspartate aminotransferase family protein CDS upstream of *astADBE* in *Pss*. In *E. coli*, this operon is activated by the transcriptional regulator NtrC in low glutamine and ammonia levels (51, 52). In *Pss,* genes encoding the two-component regulatory system NtrBC are activated; however, the *ast* operon is repressed. Previous metabolomic characterization shows that glutamine is at much greater concentrations (3.5 mM) compared to any other compound in xylem fluid (36). This suggests that *Pss* could utilize glutamine from the xylem fluid and would repress the unnecessary production of glutamine through the AST assimilation pathway.

Finally, two genes in the *Pss* Lrp regulon were associated with the repressed 4-amino-4-deoxy-alpha-L-arabinopyranosyl undecaprenyl phosphate biosynthetic process. The *arnDC* genes associated with this biological process are both found on the plasmid NZ_CP017591 within the *arnBCADTEF* operon. In *E. coli*, the 4-amino-4-deoxy-alpha-L-arabinopyranosyl undecaprenyl phosphate biosynthetic process encoded by the *arn* operon produces modified lipid A (L-Ara4N modified lipid A), which provides increased resistance to cationic antimicrobial peptides (53, 54). Lipid A is a component of lipopolysaccharide (LPS) in the outer membrane in gram-negative bacteria, and in plants, microbe-associated molecular pattern immunity is triggered by components of LPS (48, 55). *Pss* may be repressing the biosynthesis of components of LPS, like lipid A, to avoid detection by the plant host. This would complement the role of the more external EPS layer in providing protection against secreted plant defense compounds.

Other annotated Lrp-regulon genes of interest that did not fit into the biological processes described above are *cheR*, glycoside hydrolase family 10 protein CDS, and N-acetylmuramoyl-L-alanine amidase CDS (Table 1). The gene *cheR* is well known for its involvement in chemotactic response and motility (56). Additionally, adjacent *Pss* genes *cheZ* and *cheY* (involved in dephosphorylation and control of flagellar rotation, respectively) were also found to be repressed by Lrp (Table S1). Lrp has been associated with the control of bacterial motility in *Pss*, as well as in other closely related phytopathogens, like *Erwinia amylovora* (16, 19). Thus, CheR may play an important role in the movement of *Pss* within the plant.

Glycoside hydrolase family 10 proteins are involved in the degradation of major structural components in plant cell walls known as heteroxylans (57, 58). This activity may be necessary during the early stages of *Pss* infection for nutrient extraction or movement through the host vascular system but unnecessary for survival once established in the xylem (58). Finally, N-acetylmuramoyl-L-alanine amidases are widespread among bacteria and associated with cell wall modification and degradation (59). Repression of an enzyme that could compromise the protective peptidoglycan layer during infection may help afford protection to *Pss* in the xylem. Further investigation of the Lrp-regulated genes in *Pss* would enable the assignment of more precise physiological functions during *in planta* growth.

The *in vitro* analysis of the metabolism of sole carbon and nitrogen sources by wild-type *Pss* and the *∆lrp* strain offers an alternative experimental approach to examine the physiological outputs under Lrp regulation. Deletion of *lrp* had larger impacts on carbon source utilization compared to nitrogen sources for those substrates tested in the Biolog plates. However, not all of these carbon sources may be available for *Pss* to metabolize within the xylem environment. There were some parallel trends in the function of genes associated with the Lrp regulon *in planta* and the capacity of *Pss* to grow on certain nutrient sources *in vitro*. The RNA-Seq data pointed to the importance of amino acid transport and utilization, especially with regard to nitrogen metabolism and acquisition, and several amino acids were identified as being metabolized as sole nitrogen sources by *Pss* from the Biolog analysis (i.e., L-alanine, L-glutamic acid, L-methionine, L-serine, N-acetyl-D-glucosamine, Ala-Gly, Ala-Leu, and Ala-Thr). Similarly, an important role for intermediates associated with the tricarboxylic acid and glyoxylate cycles (i.e., L-malic acid, fumaric acid, succinic acid, and pyruvic acid) was observed both *in planta* and *in vitro*.

In conclusion, this study has defined genes associated with the Lrp regulon of the bacterial maize pathogen *Pss. Pss* must be able to sense its local environment and adjust its physiology to appropriately adapt and grow. Both in the maize xylem and *in vitro*, Lrp appears to be associated with the uptake and utilization of nitrogen-containing compounds. Lrp is also involved in the regulation of genes associated with protective mechanisms, including biofilm formation. What metabolites Lrp senses to elicit these responses remains unknown, but an analysis of the precise metabolites present in xylem fluid will likely provide key insights.

## MATERIALS AND METHODS

### Bacterial strains and growth medium

Isogenic *Pantoea stewartii* (*Pss*) DC283 wild-type (60) and *∆lrp* strains (16) were used for experiments. The *∆lrp* strain was shown to be successfully reverted back to wild type in prior work (16). Both strains were grown in Luria-Bertani broth (LB10 g/L tryptone, 5 g/L NaCl, and 5 g/L yeast extract) supplemented with 30 µg/mL nalidixic acid (NA).

### Growth assays in Biolog PM1 and PM3 microplates

Biolog microplates contained a negative control well and 95 sole-source carbon (PM1) or nitrogen (PM3) substrates. The microplates were prepared following the manufacturer’s protocol. However, the volumes were adjusted to prepare 10 mL of inoculating buffer for just one plate at a time. For PM1 plates, 8.33 mL of 1.2× IF-0 (Biolog), 1.57 mL of sterile water, and 0.1 mL of 100× tetrazolium indicator dye (Biolog) were combined. For PM3 plates, 8.33 mL of 1.2× IF-0 (Biolog), 1.37 mL of sterile water, 0.1 mL of 100× tetrazolium indicator dye (Biolog), and 0.2 mL of 1 M sodium succinate were combined. Overnight LB liquid cultures of *Pss* strains DC283 (wild type) or Δ*lrp* were diluted to an OD_600_ of 0.02 and grown to an OD_600_ of 0.2. Then, two 1.25 mL aliquots of culture were washed twice with an equal volume of phosphate buffered saline solution (PBS; 137 mM NaCl, 2.7 mM KCl, and 10 mM Na_2_HPO_4_, pH 7.4). Cells were centrifuged at 15,000 rpm for 5 min in an Eppendorf microcentrifuge 5424 with rotor FA-45-24-11. The washed cell pellets were resuspended in the inoculating buffer to achieve an OD_600_ of 0.05, and then 100 µL was pipetted into each well on the microplate. A BreatheEasy membrane (Diversified Biotech) was added to the top of the plate, and then the plates were incubated in a Synergy HTX multimode plate reader at 30°C for 24 h with orbital shaking, and an absorbance reading at 590 nm was taken every 20 min. Absorbance readings from the final 24 h time point for the three experimental trials were averaged, and the standard error was calculated. A *t*-test was performed using two-tailed distribution and equal variance parameters to determine significant differences (*P* < 0.05) between (i) the negative control versus each sole source carbon or nitrogen source and (ii) the wild type versus Δ*lrp* for each sole carbon or nitrogen source.

### Growth of cultures for *in planta* infection

Overnight cultures of the *Pss* DC283 and ∆*lrp* strains were grown by shaking at 30°C and 250 rpm in LB broth with 30 µg/mL NA. The overnight cultures were diluted to an OD_600_ of 0.05 in the same medium and grown to an OD_600_ of 0.2. One milliliter of culture was pelleted via centrifugation (Eppendorf centrifuge 5424, rotor FA-45-24-11) for 1 min at 10,000 rpm. The cell pellet was resuspended and washed twice in 1 mL PBS (27) prior to plant inoculation.

### Growth and inoculation of plants for RNA-Seq analysis

*Zea mays* maize plants (strain B73, 2020 seed harvest from the Mackey laboratory, Ohio, USA) were grown in Pro-Mix BX General Purpose Mycorrhizae soil. Up to four full trays (12 plants per tray) were grown in a Percival Scientific (CMP4030) plant chamber. Plant trays were filled and maintained at a level of 2 cm with Milli-Q H_2_O. Maize plants were grown at 30°C and ~80% humidity with 16-h light and 8-h dark cycles (~200 mE m^−2^ s^−1^ light intensity) for 5 days prior to inoculation. After 5 days of growth, the exterior surface of the maize stems was disinfected with 70% ethanol (EtOH) (coleoptile to first collar) prior to being inoculated. A 1 cm incision was made using a sterile needle (B-D PrecisionGlide 26 gauge) in the stem ~1 cm above the soil line. All plants were inoculated with 5 µL of the appropriate *Pss* strain by pipetting the inoculum in and out of the incision site five times. The plants were grown for an additional 3 days prior to harvesting.

### RNA extraction and RNA-Seq analysis

The stems of three maize plants were disinfected with 70% EtOH and cut at ~1 cm above the soil line and again before the first branch point of leaves, using an EtOH-disinfected razor blade. All surfaces and equipment were cleaned with RNase Away (Molecular Bio-Products). The stem was then sliced into small pieces (~0.5 cm in length) and transferred to a 15 mL conical tube containing 2 mL of RNA Protect (Qiagen). Samples were placed on ice and gently rocked for 30 min. Afterward, 1.5 mL of the liquid contents was pelleted via centrifugation (Eppendorf centrifuge 5424) for 10 min at 5,000 rpm. The supernatant was removed, and the cell pellets were stored at −80°C prior to RNA extraction.

The frozen pellets were resuspended in 100 µL RNase-free Tris-EDTA buffer containing 15 mg/mL lysozyme and 10 µL of 20 mg/mL Proteinase K (Qiagen) (27). After resuspension, 700 µL of Qiazol (Qiagen) was added to the sample, and the sample was vortexed for 10 s every 2 min at room temperature over a 10 min period. Next, 140 µL of chloroform was added to the sample, and the tube was mixed vigorously by hand. The sample was subjected to centrifugation for 15 min at 12,000 rpm (Eppendorf centrifuge 5424). Then, the aqueous phase was carefully transferred to a new collection tube. An 840 µL volume of 100% EtOH was added to the sample, and the tube was mixed vigorously by hand. The miRNeasy kit (Qiagen) with on-column DNase digestion was used for total RNA extraction as previously described (27). Extracted RNA samples were sent to the Virginia Tech Fralin Life Sciences Genomics Sequencing Center to determine RNA integrity. A minimum RNA integrity number value of 7.0 was required for the continued analysis of a sample. RNA samples were then sent to the Roy J. Carver Biotechnology Center at the University of Illinois, where ribosomal RNA was removed from samples prior to cDNA conversion, and Illumina Hi-Seq 2500 100 nucleotide (nt) single-end read RNA-sequencing provided single-end reads of high-quality, triplicate RNA samples of wild-type DC283 and *∆lrp* mutant strains grown *in planta*.

### Bioinformatic analysis of RNA-Seq data

RNA-Seq read data for the triplicate wild-type and *∆lrp* strain samples were imported into Geneious Prime software. The DC283 complete genome (21) (CP017581 to CP017592) was retrieved from the NCBI nucleotide database for use as the reference genome library. This provided 12 distinct contigs representing the fully sequenced *Pss* DC283 genome. RNA-Seq sample reads were aligned and mapped to the reference genome/contigs using an internal Geneious mapper program. RNA-Seq sample reads were either aligned to all 12 contigs of the reference genome at once (bulk alignment) or individually aligned to a single contig at a time (individual alignment), and expression levels were calculated for each assembly generated from the alignment.

Expression levels were obtained from the annotations and tracks tab for each reference sequence and all generated assemblies. The expression levels were calculated by normalizing the number of reads mapped to each coding sequence. Reads partially mapped to multiple locations (multi-mapped) were counted as partial matches (0.5 reads mapped to two locations). Expression level ratios between the DC283 and *∆lrp* alignments were calculated along with the associated *P*-values for expression level variance between alignment samples. This was completed for both individual and bulk alignment techniques, and genes with the highest and lowest expression ratios were identified. Those genes with threefold or more differential expression or greater for activated (upregulated in wild-type strain) and repressed genes (upregulated in the *∆lrp* strain) and TPM values of 100 or more were further analyzed. An exception to this was the inclusion of any coding sequence adjacent to multiple others with differential expression levels above the cutoffs. *P*-values were also used during data analysis; however, coding sequences with *P*-values greater than 0.05 were not ruled out on this criterion alone. The *P*-value signifies the magnitude of variation between the RNA-Seq data set expression levels (specifically TPM values).

### Differential gene expression analysis via DESeq2

The DESeq2 analysis was performed using Geneious Prime implementation of the R DESeq package on the RNA-Seq raw read alignments as a second method for determining differential expression between the wild-type and the *∆lrp* mutant strains. For this approach, the RNA-Seq raw read sets were aligned to single contigs (individual alignment technique) only. After aligning all six of the RNA-Seq read sets to the main chromosome (NZ_CP017581), the NZ_CP017581 reference sequence displayed the expression levels calculated for each read set. The reference sequence was then analyzed using the DESeq2 differential expression analysis to provide differential expression confidence values and *P*-values for each CDS. DC283 wild-type reads and ∆*lrp* reads were manually assigned to different sample condition groups: wild type (group A) and mutant (group B). This process was repeated for the remaining 11 chromosome reference sequences.

### Protein comparison methods

For 11 of the differentially expressed genes, chosen to be validated by qRT-PCR, the NCBI gene ID was searched and confirmed using the NCBI gene database. Next, the correlating peptide sequence was found under the NCBI reference sequences (RefSeq) for each gene. The FASTA sequence for peptides of interest from the NCBI protein database was used as a query sequence for NCBI BLASTp. Peptide sequence ID, descriptions, reference literature, and taxonomy names were collected for the 10 returns with the highest max score. NCBI gene and PubMed were used to search keywords and annotations for further evidence of related gene function in published primary literature. Finally, query coverage and percent identity were noted for the top 10 max scores provided by the NCBI BLASTp search (Table S4).

Each of the 11 *Pss* amino acid sequences of interest from the RNA-Seq analysis was also analyzed through AlphaFold to identify the predicted protein structures (28, 29). Then, each of the 11 PDB files was uploaded to the Dali database using PDB search, selecting the matches against full PDB option (30). The top 10 matches for each protein were compiled (Table S6).

### Quantitative reverse transcription PCR primer optimization

The Integrated DNA Technologies OligoAnalyzer Tool was used to generate qRT-PCR primer sets for all selected coding sequences (Table 3), and the previously identified housekeeping gene *gyrB* was used as a control (27). Primers were required to meet the following parameters: *T*_*m*_ of 62°C, GC percentage between 40% and 60%, primer length of 18–22 bp, and amplicon length of 80–130 bp. Also, primer sets were required to have a ∆*G* of −9 or lower for potential heterodimer, self-dimer, and hairpin formation.

**TABLE 3:** qRT-PCR primer sets

Name	5′–3′ primer sequence	Primer concentration (nM)	Annealing temperature (°C)
gyrB_F	GACGTGACCACGCTCAATAATTTC	800	60
gyrB_R	CGGCTCGCACATTCGTAC	800	
yjbF_F	CTGTCACGCCTCAGGAAAT	800	60
yjbF_R	TGTAACCCAGCACGACAAA	800	
lysozyme_F	GACTATCTCTCTGCCGCAAAT	800	60
lysozyme_R	TTCCCTTCGTCGCACTAATC	800	
holin_F	ATGGGTATTGTAGGTGCTGAAA	800	60
holin_R	ATGAATGCGCCGATCTCTC	800	
rutA_F	CGCCACCATTGATTCCATTTC	800	60
rutA_R	TGCCCATTTGCTCGTACTC	800	
nac_F	CCAGTCTGCGGTGATGAAT	800	60
nac_R	TGCAGAAGCGGCATAGTT	800	
ATP-BC_F	CGCAGATGAGCGTAGAAGATAA	800	60
ATP-BC_R	TGTTGACGCAGTGAATAGAGAG	800	
aceB_F	CAATACCTCGAAGCCTGGATTA	800	60
aceB_R	GATAGAGGTACGGGCGATTTC	800	
glyco-hydro_F	CTGGATGACACTGAGCGTATTA	800	60
glyco-hydro_R	CACAGGGAACCACCTGAAATA	800	
NALA-amidase_F	CATCGGAGTTGAGGTGGTTAAT	800	60
NALA-amidase_R	ACCGCTGGAGGATATTGATTG	800	

Genomic DNA was extracted from *Pss* strain DC283 using the Qiagen DNAeasy Blood and Tissue Kit to be used as the template for primer optimization and was quantified using an Implen nanophotometer. Each qPCR reaction contained the following: 12.5 µL of SYBR Green Mix (Applied Biosystem), 4.5 µL of dH_2_O, 1 µL of forward primer (800 nM), 1 µL of reverse primer (800 nM), and 1 µL of genomic DNA template at several dilutions in a total volume of 20 µL. A starting concentration of 200 ng/µL was used for the genomic DNA template, which was serially diluted in a 1:2 ratio to a final concentration of 12.5 ng/µL to generate standard curves to confirm the efficiency of each primer set.

The qPCR master mix was prepared by combining SYBR Green Mix, dH_2_O, and primers for each primer set. A 19 µL volume of the master mix was added to each appropriate well in an Applied Biosystems 96-well MicroAmp PCR reaction microplate, including a negative control (water instead of genomic DNA template added). Genomic DNA (1 µL) was added to appropriate wells following the 1:2 dilution series described above, with all reactions performed in triplicate. Applied Biosystems MicroAmp optical adhesive film was applied to the 96-well plate after all reagents were added, and the plate was centrifuged at 1,400 rpm for 2 min in a Beckman Coulter Avanti J-26 XP centrifuge (rotor: JS-5.3) to remove air bubbles. The triplicate reactions of the standard curves were amplified using an Applied Biosystems Step One Plus RT PCR thermocycler. The thermocycler was programmed as follows: 95°C for 10 min, 40 cycles at 95°C for 15 s (denaturation), and 60°C and 64°C for 1 min (annealing temperature). Each primer set was optimized to 100% ± 10% efficiency using the standard curve of diluted template.

### cDNA conversion of RNA samples

RNA isolated from a second independent set of wild-type and *∆lrp* cells grown *in planta* as described above was converted to cDNA using the High-Capacity cDNA Reverse Transcription Kit (ThermoFisher). A master mix consisting of 2 µL of 10× RT Buffer, 0.8 µL of 25× dNTP mix (100 nM), 2 µL of 10× RT random primers, 4.2 µL of nuclease-free H_2_O, and 1 µL of MultiScribe Reverse Transcriptase was prepared for each RNA sample. Ten microliters of extracted RNA was added to 10 µL of the master mix. The 20 µL reaction was then carried out in the thermocycler (Bio-Rad T100 Thermo Cycle), which was programmed as follows: 25°C for 10 min, 37°C for 120 min, 85°C for 5 min, and 4°C hold. The cDNA samples were stored at −20°C.

### qRT-PCR validation

The cDNA samples were diluted to 20 ng/µL prior to qRT-PCR. The qRT-PCR was performed as described above in triplicate, except 1.5 µL of dH_2_O and 4 µL of cDNA were added to the appropriate well. An Applied Biosystems Step One Plus RT PCR machine was programmed for 40 cycles with a denaturation temperature of 95°C for 15 s and an annealing temperature of 60°C for 1 min. If the initial average cycle threshold (Ct) values were greater than 30 (e.g., *nac, aceB,* and *yjbF* samples), the cDNA template concentration was increased to 40–50 ng/µL, and any samples that remained with a Ct value > 30 were excluded from calculations (i.e., *yjbF* was done in duplicate). A ∆∆Ct comparative analysis was completed using *gyrB* as the internal normalization control and DC283 cDNA as the reference sample. Fold gene expression was calculated using the average ∆∆Ct values and the 2^-(∆∆Ct)^ formula (Applied Biosystems).

### Gene Ontology analysis

Blast2GO software was used for Gene Ontology (GO) annotation of the *Pss* DC283 genome. GO annotations were then matched with the sequences in the RNA-Seq data, pairing differential expression and prediction GO annotations. The genes from this paired data set were separated further into genes that were threefold or more differentially expressed (either activated or repressed) between DC283 and ∆*lrp in planta* cultures. Manual annotation was completed for four activated genes with threefold or more differential expression, but without a GO annotation provided by Blast2GO using NCBI BLASTp and Geneontology.org searches (DSJ_RS03065, *yjbF*; DSJ_RS03070, *yjbE*; DSJ_RS03060, *gfcC/yjbG*; and DSJ_RS03055, *yjbH*). Gene set enrichment was conducted on the threefold or greater differentially expressed and GO or manually annotated genes. The analysis focused on groups of genes enriched for Biological Process gene ontologies. Fisher’s exact test was utilized for statistical analysis, and *P*-values were false discovery rate adjusted. Gene groups with *P*-values of 0.05 or lower were considered to be significantly regulated.

## Data Availability

The RNA-Seq read data including an Excel file summarizing the total read counts for each sample using the *Pss* DC283 complete genome (CP017581 to CP017592) from NCBI (21) were deposited to the NCBI Sequence Read Archive (SRA) with accession numbers SAMN41777979–SAMN41777984.

## SUPPLEMENTAL MATERIAL

Figure S1 aem.00853-25-s0001.docx Venn diagram depicting differential gene expression between the bulk alignment and individual alignment methods for RNA-Seq analysis.

Table S1 aem.00853-25-s0002.xlsx Bulk alignment of RNA-Seq data for Lrp-activated and -repressed genes (3-fold or more).

Table S2 aem.00853-25-s0003.xlsx Individual alignment of RNA-Seq data for Lrp-activated and -repressed genes (3-fold or more).

Table S3 aem.00853-25-s0004.xlsx Lrp-activated and -repressed genes calculated using DeSeq2 (confidence value 3 or more).

Table S4 aem.00853-25-s0005.xlsx NCBI BLASTp alignments for select Lrp-regulated gene products.

Table S5 aem.00853-25-s0006.xlsx AlphaFold and Dali predicted structure and function analysis for select Lrp-regulated gene products.

Table S6 aem.00853-25-s0007.xlsx GO annotations.

Table S7 aem.00853-25-s0008.docx Biolog carbon and nitrogen sources used by wild-type *Pss* significantly more than the negative control, arranged from highest absorbance to lowest.

ASM does not own the copyrights to Supplemental Material that may be linked to, or accessed through, an article. The authors have granted ASM a non-exclusive, world-wide license to publish the Supplemental Material files. Please contact the corresponding author directly for reuse.
