# Supplementary material for: The role of Pantoea stewartii subsp. stewartii leucine-responsive regulatory protein (Lrp) during maize xylem growth
Source: Appl Environ Microbiol. 2025 Jun 5;91(7):e00853-25. doi: 10.1128/aem.00853-25 (PMC12285263; doi:10.1128/aem.00853-25)
Supplement: Figure S1 — Venn diagram depicting differential gene expression between the bulk alignment and individual alignment methods for RNA-Seq analysis. [file aem.00853-25-s0001.docx]

**
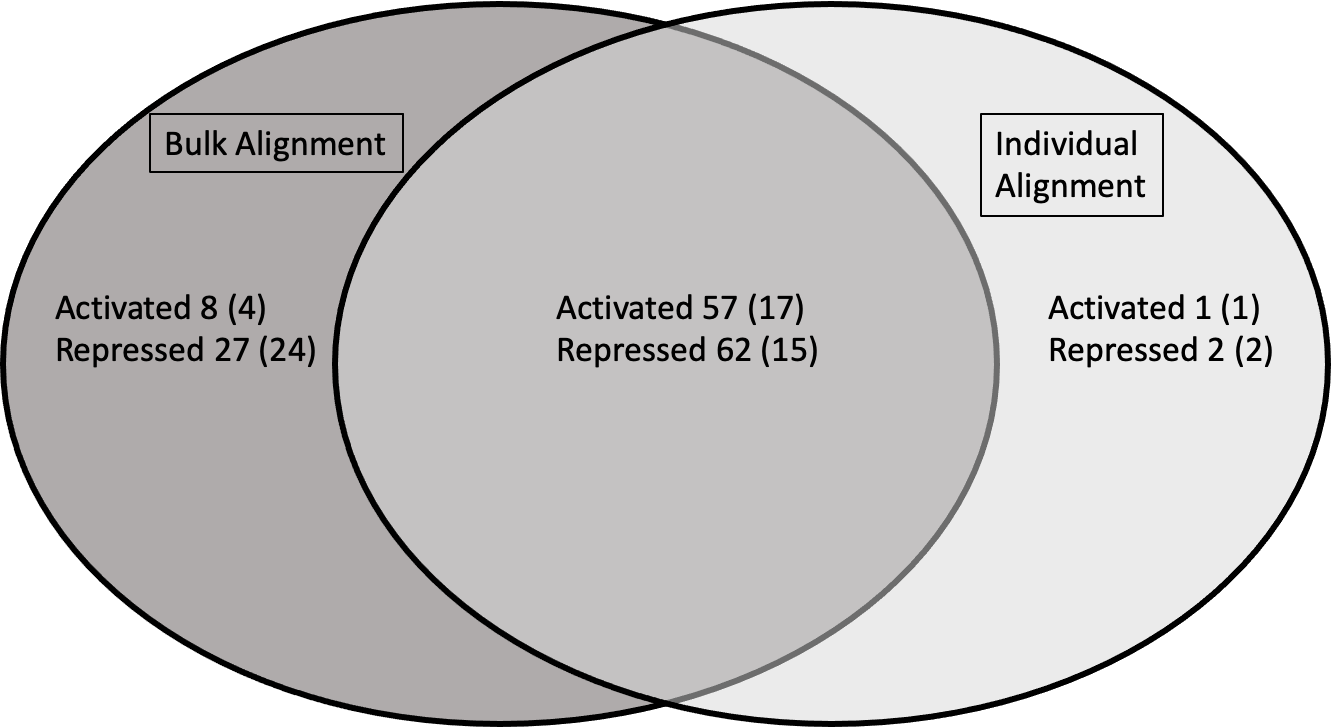
**

**Figure S1. Venn diagram depicting differential gene expression between the bulk alignment and individual alignment methods for RNA-Seq analysis.** The first values provided reflect the total number of activated or repressed genes with three-fold or more differential expression, not including any TPM cutoff. The numbers in parentheses indicate the total number of activated or repressed genes with three-fold or more differential expression and 100 or more TPM; only genes with 100 or more TPM counts in both the bulk and individual alignments were included for the overlap counts. Overall, the values were 65 (21) activated genes and 89 (56) repressed genes for only the bulk alignment and 58 (32) activated genes and 64 (37) repressed genes for only the individual alignment.
