# Supplementary material for: The role of Pantoea stewartii subsp. stewartii leucine-responsive regulatory protein (Lrp) during maize xylem growth
Source: Appl Environ Microbiol. 2025 Jun 5;91(7):e00853-25. doi: 10.1128/aem.00853-25 (PMC12285263; doi:10.1128/aem.00853-25)
Supplement: Table S7 — Biolog carbon and nitrogen sources used by wild-type Pss significantly more than the negative control, arranged from highest absorbance to lowest. [file aem.00853-25-s0008.docx]

**Table S7. Biolog carbon and nitrogen sources used by wild-type *Pss* significantly more than the negative control, arranged from highest absorbance to lowest**

| **Carbon source** | **DC283 average** | **DC283 STE** | **T-test** | **Nitrogen source** | **DC283 average** | **DC283 STE** | **T-test** |
| --- | --- | --- | --- | --- | --- | --- | --- |
| D-Glucose-6-phosphate | 2.165 | 0.036 | 2.9E-06 | Adenosine | 1.844 | 0.096 | 3.1E-04 |
| L-Arabinose | 2.109 | 0.025 | 8.8E-07 | Cytidine | 1.842 | 0.082 | 1.7E-04 |
| L-Glutamine | 2.077 | 0.037 | 3.8E-06 | Ala-Glu | 1.816 | 0.1 | 4.0E-04 |
| L-Asparagine | 2.053 | 0.049 | 1.2E-05 | Ala-Gln | 1.752 | 0.027 | 3.1E-06 |
| myo-Inositol | 2.039 | 0.066 | 3.8E-05 | Gly-Asn | 1.722 | 0.018 | 8.0E-07 |
| α-Methyl-D Galactoside | 1.907 | 0.031 | 3.5E-06 | Ala-Asp | 1.711 | 0.037 | 1.3E-05 |
| D-Xylose | 1.891 | 0.04 | 9.6E-06 | L-Methionine | 1.673 | 0.075 | 2.3E-04 |
| D-Gluconic acid | 1.888 | 0.045 | 1.4E-05 | Ala-Leu | 1.652 | 0.01 | 1.3E-07 |
| L-Serine | 1.873 | 0.074 | 1.1E-04 | Gly-Gln | 1.645 | 0.039 | 2.1E-05 |
| D-Mannose | 1.872 | 0.028 | 2.9E-06 | Ala-Gly | 1.629 | 0.029 | 6.7E-06 |
| D-Fructose-6- Phosphate | 1.844 | 0.039 | 1.0E-05 | D-Glucosamine | 1.618 | 0.159 | 5.0E-03 |
| D-Ribose | 1.827 | 0.067 | 8.6E-05 | L-Serine | 1.584 | 0.048 | 6.0E-05 |
| D-Glucose-1- Phosphate | 1.817 | 0.038 | 9.9E-06 | N-Acetyl-D Glucosamine | 1.521 | 0.015 | 1.0E-06 |
| Fumaric Acid | 1.799 | 0.045 | 2.1E-05 | L-Glutamine | 1.507 | 0.06 | 2.1E-04 |
| D-Mannitol | 1.781 | 0.051 | 3.6E-05 | Ala-His | 1.46 | 0.01 | 3.2E-07 |
| D-Fructose | 1.777 | 0.033 | 7.3E-06 | L-Asparagine | 1.432 | 0.045 | 9.7E-05 |
| D-Galactose | 1.77 | 0.051 | 3.5E-05 | Guanine | 1.431 | 0.122 | 4.5E-03 |
| N-Acetyl-D Glucosamine | 1.745 | 0.068 | 1.2E-04 | Gly-Met | 1.403 | 0.051 | 1.9E-04 |
| D-Melibiose | 1.74 | 0.035 | 9.9E-06 | Met-Ala | 1.358 | 0.015 | 2.5E-06 |
| Pyruvic Acid | 1.73 | 0.063 | 9.4E-05 | L-Alanine | 1.339 | 0.075 | 1.2E-03 |
| Succinic acid | 1.678 | 0.044 | 3.0E-05 | Allantoin | 1.329 | 0.027 | 2.4E-05 |
| L-Malic Acid | 1.673 | 0.051 | 5.3E-05 | L-Aspartic Acid | 1.327 | 0.066 | 8.3E-04 |
| Glycerol | 1.651 | 0.131 | 2.1E-03 | L-Glutamic Acid | 1.271 | 0.031 | 6.8E-05 |
| D,L-Malic Acid | 1.605 | 0.063 | 1.6E-04 | Uric Acid | 1.23 | 0.051 | 6.3E-04 |
| L-Glutamic Acid | 1.475 | 0.067 | 3.7E-04 | Gly-Glu | 1.116 | 0.072 | 5.8E-03 |
| Adenosine | 1.437 | 0.15 | 8.9E-03 | Ala-Thr | 1.088 | 0.021 | 8.1E-05 |
| ß-Methyl-D Glucoside | 1.383 | 0.035 | 5.7E-05 | Adenine | 1.026 | 0.016 | 6.2E-05 |
| α-D-Glucose | 1.381 | 0.058 | 3.6E-04 | Ammonium Formate | 0.998 | 0.038 | 2.2E-03 |
| Sucrose | 1.331 | 0.021 | 1.4E-05 | D-Asparagine | 0.88 | 0.016 | 9.2E-04 |
| L-Alanyl-Glycine | 1.314 | 0.033 | 6.7E-05 | Urea | 0.846 | 0.018 | 3.6E-03 |
| D-Trehalose | 1.284 | 0.064 | 9.8E-04 | Glycine | 0.845 | 0.01 | 4.2E-04 |
| L-Alanine | 1.253 | 0.038 | 1.8E-04 | D-Alanine | 0.824 | 0.003 | 9.0E-05 |
| Glycyl-LGlutamic Acid | 1.206 | 0.054 | 9.2E-04 | Parabanic Acid | 0.817 | 0.028 | 3.6E-02 |
| D-Malic Acid | 1.195 | 0.065 | 2.1E-03 | Xanthine | 0.805 | 0.022 | 2.8E-02 |
| Methyl Pyruvate | 1.09 | 0.092 | 1.7E-02 | Cytosine | 0.777 | 0.013 | 2.8E-02 |
| Glycyl-L-Aspartic Acid | 1.085 | 0.057 | 3.5E-03 | Negative Control | 0.729 | 0.005 | NA |
| Bromo Succinic Acid | 1.036 | 0.022 | 2.1E-04 |  |  |  |  |
| L-Aspartic acid | 1.014 | 0.027 | 5.6E-04 |  |  |  |  |
| m-Tartaric Acid | 0.962 | 0.034 | 2.5E-03 |  |  |  |  |
| Glycyl-L-Proline | 0.941 | 0.045 | 9.5E-03 |  |  |  |  |
| Citric Acid | 0.802 | 0.014 | 1.1E-02 |  |  |  |  |
| Negative Control | 0.723 | 0.011 | NA |  |  |  |  |
